# GFAP positivity in neurons following traumatic brain injuries

**DOI:** 10.1007/s00414-021-02568-1

**Published:** 2021-06-11

**Authors:** Johann Zwirner, Julia Lier, Heike Franke, Niels Hammer, Jakob Matschke, Florian Trautz, Rexon Tse, Benjamin Ondruschka

**Affiliations:** 1 Department of Anatomy, University of Otago, Dunedin, New Zealand; 2 Institute of Legal Medicine, University Medical Center Hamburg-Eppendorf, Hamburg, Germany; 3 Institute of Legal Medicine, University of Leipzig, Leipzig, Germany; 4 Institute of Anatomy, University of Leipzig, Leipzig, Germany; 5 Rudolf Boehm Institute of Pharmacology and Toxicology, University of Leipzig, Leipzig, Germany; 6 Institute of Macroscopic and Clinical Anatomy, University of Graz, Graz, Austria; 7 Department of Trauma, Orthopedic and Plastic Surgery, University Hospital of Leipzig, Leipzig, Germany; 8 Fraunhofer Institute for Machine Tools and Forming Technology, Dresden, Germany; 9 Institute of Neuropathology, University Medical Center Hamburg-Eppendorf, Hamburg, Germany; 10 Department of Forensic Pathology, LabPLUS, Auckland City Hospital, Auckland, New Zealand

**Keywords:** Glial fibrillary acidic protein, Immunofluorescence, Immunohistochemistry, Neuron, Traumatic brain injury

## Abstract

Glial fibrillary acidic protein (GFAP) is a well-established astrocytic biomarker for the diagnosis, monitoring and outcome prediction of traumatic brain injury (TBI). Few studies stated an accumulation of neuronal GFAP that was observed in various brain pathologies, including traumatic brain injuries. As the neuronal immunopositivity for GFAP in Alzheimer patients was shown to cross-react with non-GFAP epitopes, the neuronal immunopositivity for GFAP in TBI patients should be challenged. In this study, cerebral and cerebellar tissues of 52 TBI fatalities and 17 controls were screened for immunopositivity for GFAP in neurons by means of immunohistochemistry and immunofluorescence. The results revealed that neuronal immunopositivity for GFAP is most likely a staining artefact as negative controls also revealed neuronal GFAP staining. However, the phenomenon was twice as frequent for TBI fatalities compared to non-TBI control cases (12 vs. 6%). Neuronal GFAP staining was observed in the pericontusional zone and the ipsilateral hippocampus, but was absent in the contralateral cortex of TBI cases. Immunopositivity for GFAP was significantly correlated with the survival time (*r* = 0.306, *P* = 0.015), but no correlations were found with age at death, sex nor the post-mortem interval in TBI fatalities. This study provides evidence that the TBI-associated neuronal immunopositivity for GFAP is indeed a staining artefact. However, an absence post-traumatic neuronal GFAP cannot readily be assumed. Regardless of the particular mechanism, this study revealed that the artefact/potential neuronal immunopositivity for GFAP is a global, rather than a regional brain phenomenon and might be useful for minimum TBI survival time determinations, if certain exclusion criteria are strictly respected.

## Introduction

Traumatic brain injury (TBI) is defined as a chronic disease process [1] that contributes considerably to the global burden of injury [2]. Traumatic impact to the head causes glial, neuronal and axonal injuries followed by neuroinflammation and an activation of microglia [3–5]. The resulting brain damage and the subsequent recovery are indirectly reflected by an increase or decrease in intracellular proteins within brain tissue or other organic compounds within particular body fluids, which are, therefore, deemed as cerebral biomarkers [6]. Some common examples of TBI-related biomarkers are neuron-specific enolase, brain-derived neurotrophic factor, tau protein, neurofilament, myelin basic protein, S100 calcium-binding protein B (S100B), interleukin 6 (IL-6) and glial fibrillary acidic protein (GFAP) [3, 5, 7–11].

In a post-mortem setting, TBI-related biomarkers can be investigated within the brain to explore the pathophysiological characteristics of TBI. Alternatively, these biomarkers may be utilized to establish new forensic methods for estimating survival time and time since death. However, only few forensic studies have systematically investigated TBI-related biomarkers within human brain tissue [5, 12–16], to date. As a result, little is known about the post-traumatic pathophysiology of these markers at a cellular level. Surprisingly, previous studies from our group observed S100B within neurons following TBI after a minimum survival time of 2 h, a marker which is usually known to be specific for glial cells (astrocytes, oligodendrocytes) [12, 13]. It is unclear whether neurons synthesize glia-specific proteins following the traumatic brain injury or whether these proteins enter the neuron via an active or passive transport pre-mortem or post-mortem. Moreover, the aforementioned observations of S100B raise the question whether other TBI-related biomarkers that are normally not known as neuron specific or even neuronally expressed can also be detected in neurons following the traumatic brain injury.

GFAP is the major protein component of glial intermediate filaments, which substantially increases within astrocytes in response to a traumatic brain injury [17]. Clinically, increased blood levels of GFAP were shown to be a well-suited biomarker to discriminate TBIs from controls [18, 19]. Detection of GFAP in post-mortem serum [9, 10] and cerebrospinal fluid [10] samples is also a suitable method to discriminate between lethal TBIs and controls. Both clinically and forensically, the potential to discriminate TBIs from controls was further improved when GFAP was used in combination with other biomarkers such as ubiquitin C-terminal hydrolase-L1 [20] or IL-6 [21].

GFAP-positive staining of human neurons following traumatic brain injury was previously observed in the CA2, CA3 and CA4 regions of the hippocampus in subacute and delayed deaths [22]. Furthermore, an increase in GFAP expression in rodent hippocampal neurons was shown after TBI using single-cell RNA sequencing [23]. However, it remains unclear to date whether the neuronal positivity for GFAP is an exclusive observation for the hippocampus or also affects other brain regions. In light of previous observations of cross-reactions between GFAP antibodies and epitopes of neurofilament-L in Alzheimer’s disease (AD) patients [24], this given study will particularly focus on the possibility that neuronal GFAP is a processing artefact, rather than a reactive process following brain injuries.

## Material/methods

### Retrieval of human brain tissue samples

This study re-evaluated digitalized photographs of brain tissue samples taken from 50 TBI fatalities and 16 controls collected for our study on astrocytic GFAP expression following traumatic brain injuries (for individual case details see Trautz et al. [5]). In brief, human brain samples were retrieved during forensic autopsies; afterwards, they were submerged in neutral buffered 4% formaldehyde, which was changed once after 24 h. The duration of fixation was up to 3 weeks before embedding in paraffin. The control samples were collected from individuals who died of sudden natural cardiovascular causes of death with absence of acute or former injuries of the central nervous system. To investigate a potential region dependency of immunopositivity for GFAP in neurons within the brain in TBI fatalities, samples from the following four brain sites were evaluated: (i) the cerebral area (cortex and adjacent white matter) next to the either microscopically (haemorrhages with abundant necrotic tissue) or macroscopically verified contusions, (ii) a cerebral region (cortex and adjacent white matter) opposite to the contusion called the contralateral cortex (CLC), which was microscopically and macroscopically free of contrecoup haemorrhages, (iii) the CA4 region of the hippocampus (HC) and (iv) the cerebellum (CB). Hippocampal and cerebellar samples were taken from regions ipsilateral to the cerebral contusion as also performed previously [5, 12, 15].

Using semiquantitative analyses, the contusions were located in the prefrontal, temporoparietal and occipital regions of the brain in 22, 23 and 5 TBI fatalities, respectively. For controls, samples were retrieved from the following brain regions, which varied from TBI fatalities as no impact zone was present: (i) the frontal cortex and adjacent white matter (FC), (ii) the ipsilateral CA4 region of the HC and (iii) the ipsilateral CB.

In line with previous studies [5, 12], the TBI cases were further categorized into the following three groups according to their survival time following the traumatic event: an acute group (survival time < 2 h), a subacute group (survival time 2–72 h) and a delayed group (survival time > 72 h). The Ethics Committee of the University of Leipzig, Germany approved the study (local number 117–12-23,012,012).

### Histology, immunohistochemistry and assessment

The brain samples allocated to the subgroups and their respective sample characteristics are depicted in Table 1. Routine H&E stains were available to assess the overall morphology and potential trauma-related or inflammatory changes. The immunolabelled slices were stained with a polyclonal rabbit GFAP antibody (catalogue number Z0334; Agilent Dako, Santa Clara, USA), dilution 1:4000, performed with a fully automated immunostainer (Benchmark XT; Roche, Basel, Switzerland) using DAB as standard chromogen. For each brain region per case, a set of ten coherent “high-power field” (HPF) images was assessed, resulting in a standardized investigation area of 2.37 mm^2^ per region [25]. The commercially available object counting application SIGMA (open source, developed by Karen Grigoryan) was used to evaluate the slides in all ten digital HPF images at × 200. Only cells with a distinct cellular immunostaining which were identified as neuronal in origin were counted to ensure a repeatability of the results. Here, nucleus morphology, nucleolus location and cell boarders were used to differentiate neurons from non-neuronal cells as established morphological criteria. After immunopositivity for GFAP was noted in the neurons, the consecutive slides were stained analogously per immunolabelling and counterstained with haematoxylin.

**Table 1:** The case characteristics for the 50 TBI fatalities and 16 controls (sudden cardiovascular fatalities) that were included in the semiquantitative analysis of GFAP positivity in neurons in this study. Note that no survival time was assumed for control cases due to the immediate death resulting from the fatal cardiovascular event. *H* hours, *min* minutes

	Sex		Age		Survival time		PMI	
Group	Female	Male	Range	Median	Range	Median	Range	Median
TBI fatalities								
Acute death	7	17	18–75 years	44 years	5–90 min	15 min	5–117 h	43 h
Subacute death	4	11	23–85 years	62 years	5–50 h	2 h	31–144 h	72 h
Delayed death	2	9	26–81 years	57 years	3–144 days	11 days	22–120 h	71 h
Control group (sudden cardiovascular fatalities)								
Control	7	9	27–91 years	64 years	0 h	0 h	26–139 h	60 h

The overall staining intensity of the section was rated according to the scoring system of Remmele and Stegner [26] to relate the observed results of this study to the staining quality of the brain tissue. The scoring system implemented the following grades: (0) absence of noticeable staining, (1) slight staining with the marker being predominantly concentrated on cell bodies, (2) moderate staining with incidental spots that are more intensively coloured and some blunt processes and (3) strong staining with predominantly condensed central granulations and widespread processes. The final grade was generated by the arithmetic mean of the ten sections available per region.

### Immunofluorescence

To confirm or reject the immunohistochemical observations, an additional staining technique was performed employing immunofluorescence labelling on brain tissue of two additional TBI fatalities and one control case, which were not included in the dataset of Trautz et al. [5]. Both TBI cases were male, and were 18 and 32 years old at death. Both cases had a survival time of less than 10 min and post-mortem intervals of under 48 h. The control case was a 50-year-old male who died of an acute cardiac arrest. The procedure was performed twice using primary and secondary antibodies of different batches and manufacturers to exclude that the observations were attributed to a specific batch (or manufacturer) of antibodies (Table 2). Also, negative controls were made concomitantly to test for unspecific binding. Whereas the Agilent Dako GFAP antibody “reacts strongly with human GFAP”, the BD Pharmingen anti-GFAP cocktail “specifically detects GFAP and does not cross-react with other intermediate filaments” according to the details provided by both manufacturers. Since antibody “cocktails” are composed of monoclonal antibodies combining several targets, one could describe them as oligoclonal antibodies, combining the specificity of monoclonal and the sensitivity of polyclonal antibodies.

**Table 2:** The characteristics of the primary and secondary antibodies for the immunofluorescence are depicted. The immunofluorescence stains of GFAP and NeuN were performed twice (*) using different antibody batches

	Dilution	Host	Company (headquarter, state, country)	Catalogue number	Experiment number^*^
Primary antibodies					
GFAP	1:400	Rabbit	Agilent Dako (Santa Clara, CA, USA)	Z0334	1
GFAP	1:400	Mouse	BD Pharmingen Inc (San Diego, CA, USA)	556,330	2
NeuN	1:500	Mouse	Millipore Sigma (Burlington MA, USA)	MAB377	1
NeuN	1:500	Rabbit	Millipore Sigma (Burlington MA, USA)	ABN78	2
Secondary antibodies					
Alexa Fluor 488, anti-rabbit	1:200	Donkey	Invitrogen, Thermo Fisher, Scientific (Waltham, MA, USA)	A21206	1
Alexa Fluor 488, anti-mouse	1:200	Donkey	Invitrogen, Thermo Fisher, Scientific (Waltham, MA, USA)	A21202	1
Alexa Fluor 568, anti-rabbit	1:200	Donkey	Invitrogen, Thermo Fisher, Scientific (Waltham, MA, USA)	A10042	2
Alexa Fluor 568, anti-mouse	1:200	Donkey	Invitrogen, Thermo Fisher, Scientific (Waltham, MA, USA)	A10037	2

NeuN was used as a typical and specific neuronal marker of human brain tissue [27].

The tissue was cut into 12-μm-thick serial sections. Antigen retrieval was achieved using microwave pre-treatment in citrate buffer (pH = 6.0, 98 °C, 2 × 20 min). Tissues were washed and then blocked with 1% bovine serum albumin (BSA; A7906, Sigma-Aldrich, St. Louis, USA) in PBS-Triton (0.03%, PBS-T) to prevent further non-specific binding. Then, sections were incubated with the primary antibodies overnight in an adequate dilution in 0.5% BSA in PBS-T at 4 °C (Table 2). For the negative controls, the primary antibody was omitted and the section was incubated in 0.5% BSA in PBS-T overnight. After rinsing with PBS-T, the Alexa Fluor 488 and Alexa Fluor 568-conjugated secondary antibodies were applied and the slices were incubated for 60 min at room temperature at a dilution of 1:200. Counterstaining with 4′,6-diamidino-2-phenylindole dihydrochloride (DAPI, dilution 1:50.000; Invitrogen, Waltham, MA, USA) was done to detect cell nuclei. Slices were then washed and cover slipped. Images were taken using an Olympus BX40 fluorescent microscope (Olympus Deutschland, Hamburg, Germany). Due to the young age of the tested individuals, no lipofuscin quenching was performed to prevent a decrease of staining intensity.

### Statistical analyses

Data analysis was conducted using Prism version 8 (GraphPad Software, La Jolla, CA, USA) and Microsoft Excel version 16.16 (Microsoft Corporation, Redmond, WA, USA). The Shapiro–Wilk test was used to test the Gaussian distribution of the data. Pearson’s *r* and Spearman’s *ϱ* were reported for parametric and non-parametric data, respectively. The occurrence of immunopositivity for GFAP in neurons of any brain region per cadaver was correlated with age at death, sex, survival time and PMI. The total number of immunopositivity for GFAP in neurons was correlated with the total number of GFAP-positive astrocytes of the respective brain region (metadata in Trautz et al. [5]) and the staining score mentioned above. *P*-values of 0.05 or less were considered to be statistically significant.

## Results

### Immunopositivity for GFAP in neurons was detected in TBI cases and controls and this correlated with the survival times in TBI cases

Immunopositivity for GFAP in neurons in any of the investigated brain regions was detected in 6 out of 50 TBI cases (12%), as well as 1 (44-year-old male who died of an acute coronary insufficiency) out of 16 controls (6%). A significant and moderately positive correlation was observed between the survival time and the occurrence of immunopositivity for GFAP in neurons in any of the four investigated sub-regions from the TBI cases (*r* = 0.306, *P* = 0.015). The occurrence of immunopositivity for GFAP in neurons in any of the sub-regions did not correlate with age at death (*r* =  − 0.016, *P* = 0.451), sex (*r* =  − 0.013, *P* = 0.459) and PMI (*r* = 0.011, *P* = 0.467) on a statistically significant level for TBI cases. Age, sex and PMI correlations were not performed for control cases due to the limited sample size.

### Immunopositivity for GFAP in neurons in TBI cases was observed in the pericontusional zone and the ipsilateral hippocampus, but not the contralateral cortex

**Fig. 1:**
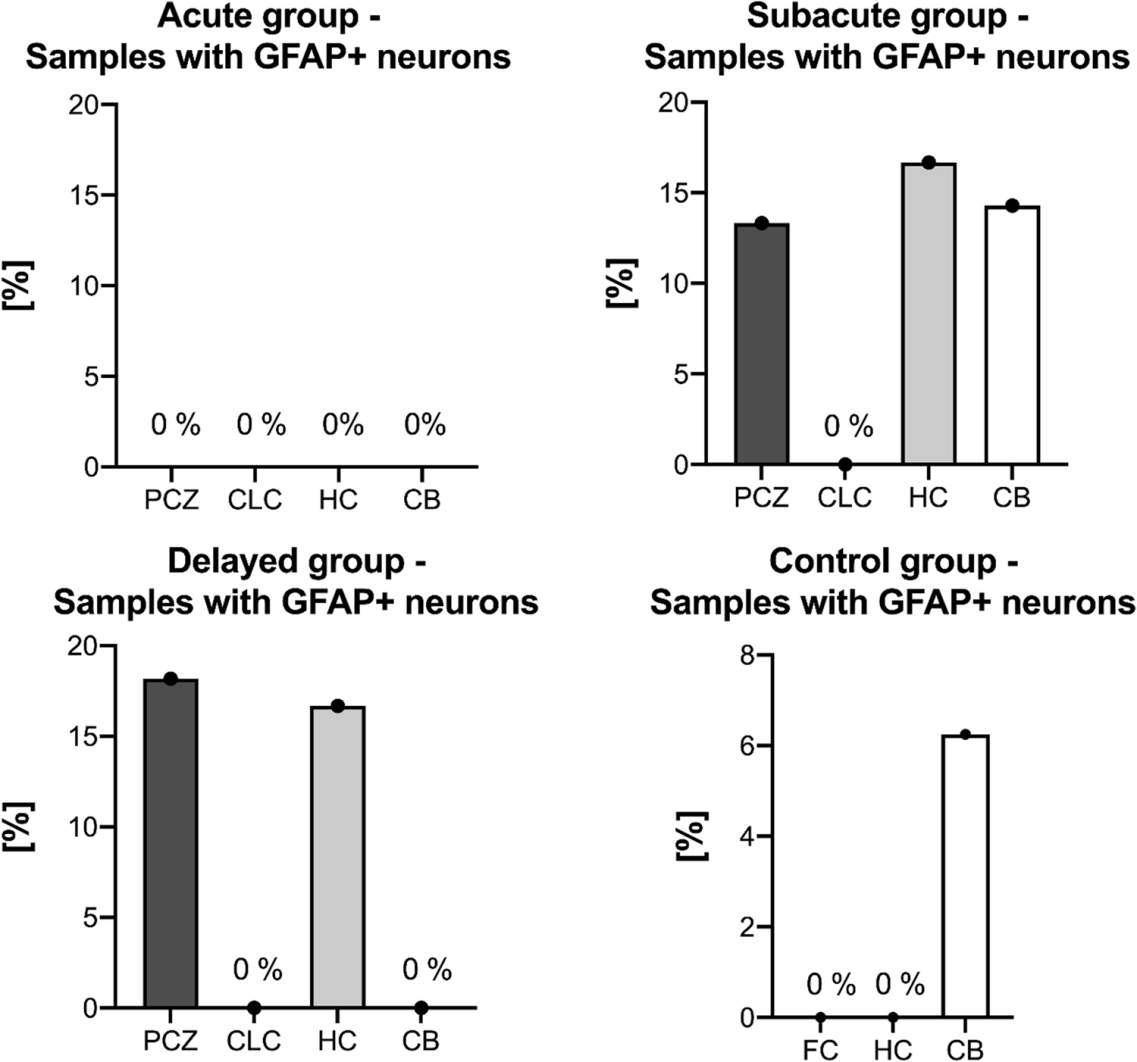
The percentage of GFAP positivity (GFAP +) in neurons per investigated sub-region is separately depicted for the different prolonged TBI survival times and the control group. CB cerebellum, CLC contralateral cortex, FC frontal cortex, HC hippocampus, PCZ pericontusional zone

Immunopositivity for GFAP in neurons was observed in the PCZ and HC of 13 and 17% of the subacute TBI fatalities (Fig. 1). Similarly, immunopositivity for GFAP in neurons was found in 17 and 18% of the HC and PCZ in delayed TBI fatalities, respectively (Fig. 1). The CB contained immunopositivity for GFAP in neurons in 14% of subacute TBI fatalities (Fig. 1). None of the acute TBI cases or CLC regions of subacute and delayed TBI fatalities revealed any immunopositivity for GFAP in neurons (Fig. 1). Control samples showed immunopositivity for GFAP only in Purkinje neurons in 6% of cerebellar samples (Fig. 1). The two TBI samples that presented immunopositivity for GFAP in Purkinje neurons had a contusion in the prefrontal cortex area. Immunopositivity for GFAP in neurons was found in the HC following both temporal contusions (*n* = 2) and a prefrontal cortical contusion (*n* = 1). No immunopositivity for GFAP in neurons was detected in the frontal cortices or hippocampi of controls.

### The absolute number of GFAP-labelled neurons per sub-region is low

**Fig. 2:**
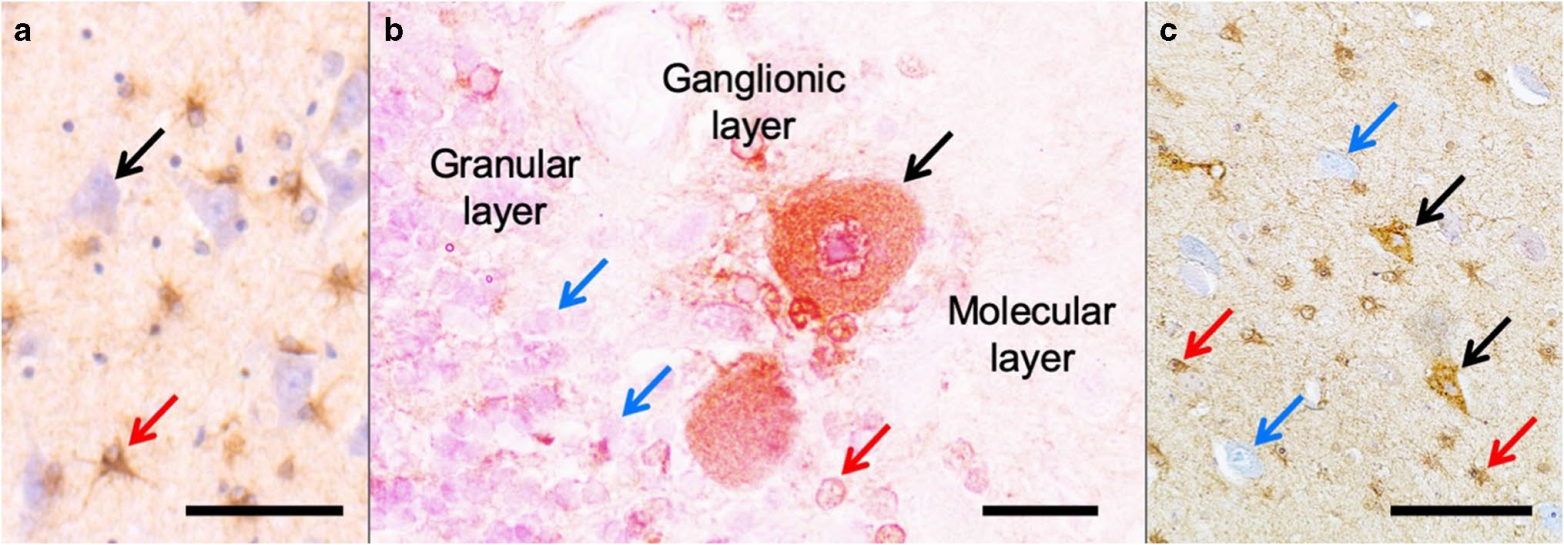
GFAP positivity in neurons is depicted in different regions of the brain. **a** Counterstaining was conducted with blueing reagent, which visualizes the neurons (black arrow). GFAP-positive astrocytes are depicted (red arrow). **b** In the cerebellar cortex, immunopositivity for GFAP is depicted for Purkinje neurons (black arrow) and Bergmann glial cells (red arrow). The granular layer contains abundant GFAP-negative granular cells (blue arrows). **c** The GFAP positivity in several neurons (black arrows) of the cerebral cortex is accompanied by GFAP-positive astrocytes (red arrows) and GFAP-negative neuronal cell bodies (blue arrows) in the anti-GFAP immunolabelling. Scale bars: 100 μm (**a**), 25 μm (**b**) and 100 μm (**c**)

The low absolute number of GFAP-labelled neurons correlated only weakly and insignificantly with the number of GFAP-positive astrocytes of the respective HPF (*r* = 0.247, *P* = 0.244). Also, the adapted Remmele and Stegner staining score did not influence the absolute number of GFAP-labelled neurons significantly (*r* = 0.086, *P* = 0.410). Figure 2 shows typical GFAP-positive astrocytes, as well as immunopositivity for GFAP in representative neuronal cells in the anti-GFAP immunolabellings that were evaluated in this study.

### Immunofluorescence stains reveal non-specific binding of the secondary antibody

**Fig. 3:**
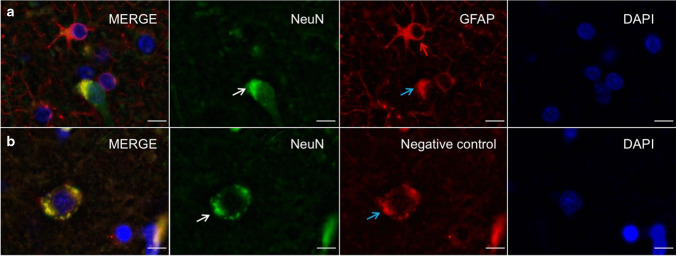
Immunofluorescence images of the cerebral cortex of a 32-year-old donor are depicted. **a** A neuron is stained with anti-NeuN (white arrow) immunolabelling. The anti-GFAP immunolabelling shows GFAP-positive astrocytes (red arrow), as well as GFAP-positive staining within the NeuN-positive neuron (NeuN is predominantly a nuclear marker). **b** The negative control for the anti-GFAP immunolabelling reveals a non-specific binding of the secondary antibody to the deposits (blue arrow) within a NeuN-positive neuron (white arrow). Scale bars 10 µm

**Fig. 4:**
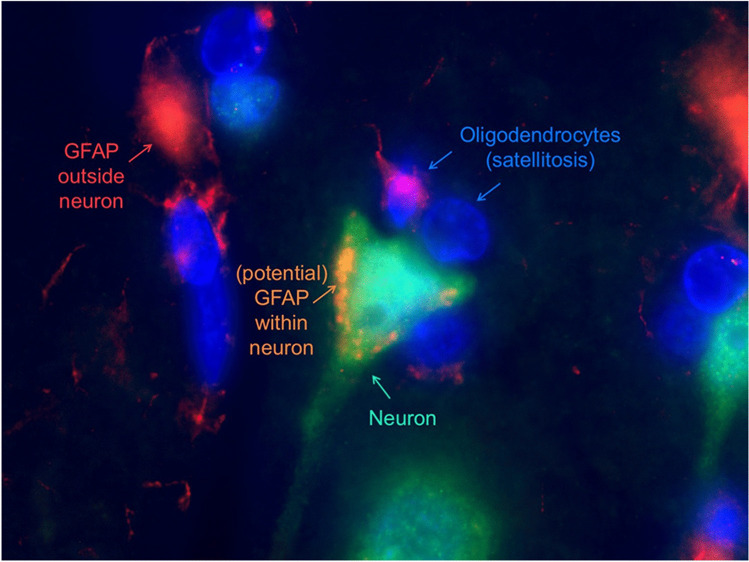
An immunofluorescence image of the cerebral cortex is depicted. Deposits representing either GFAP or a non-specific binding of the secondary antibody are visible within the cytoplasm of a neuron of a 30-year-old male. An abnormal cluster of oligodendrocytes (satellitosis) is depicted around the neurons as a sign of potential neuronal damage

The double immunofluorescence stains with primary anti-GFAP and anti-NeuN antibodies revealed GFAP-positive staining within vital (DAPI-positive) neuronal cell bodies, which are also positively labelled with NeuN (Fig. 3a). However, a comparable, more peripheral and granular reaction of the secondary antibody was observed in the negative control (Fig. 3b). These positive stainings were present even when another antibody batch was used for both the primary and secondary antibodies, respectively. Furthermore, the GFAP-positive stainings were detected for both of the secondary antibodies used in this study (Fig. 4). However, comparable GFAP-positive stainings were also present in the 488-channel for the negative control.

## Discussion

Traumatic brain injuries were shown to impact cell type-specific genes and additionally alter the genomic co-expression across cell types [23]. With regard to GFAP, a TBI-related neuronal deposition was observed previously and was suspected to be induced by the traumatic event [22]. Additionally, the former study suggested that the distinct immunopositivity for GFAP could highlight a GFAP-related protein, rather than actual GFAP positivity [22]. Moreover, neuronal GFAP staining was observed in AD patients [28], which further substantiates that neuronal GFAP might be related to neuron degeneration or damage, thereby corroborating the findings in TBI cases [22]. However, the immunopositivity for GFAP in AD patients was shown to be related to a cross-reactivity of the GFAP antibody with neurofilament-L [24]. This was attributed to a homology between amino acids of the GFAP^+1^ peptide and the tail domain of neurofilament-L [24]. Therefore, the current study not only performed a standard negative control, but also confirmed the findings applying another antibody batch and even an additional immunofluorescence stain. The results of this study suggest the neuronal immunopositivity for GFAP is a processing artefact, rather than an actual reactive neuronal occurrence of GFAP post-TBI. With respect to the previous findings of neuronal immunopositivity for GFAP and the fact that this given study cannot entirely prove the absence of neuronal GFAP, factors for and against a neuronal staining of GFAP will be discussed separately below.

### Indicators and explanations for an absence of neuronal GFAP

Even though remarkable evidence exists that supports the neuronal potential to synthesize GFAP mRNA [23, 28], it has to be considered that the GFAP immunoreaction might solely be an artefact and, therefore, not truly depict neuronal GFAP. Regarding the former, the strongest argument in this given study forms the observation of fluorescent neuronal staining in the negative control of the anti-GFAP stain. The immunopositivity of the anti-GFAP-negative control suggests a non-specific binding of the secondary antibody, which contradicts the observation of neuronal GFAP. Regarding this, Middeldorp and colleagues were able to show that a neuronal staining with different GFAP antibodies was caused by a cross-reaction with neurofilament-L (NF-L). This is most likely due to a homology of the GFAP peptide with several amino acids in the tail domain of NF-L [24]. Even though the antibodies used are described to have increased specificity in epitope binding, the exact epitope the antibody was binding to is unknown. Therefore, it is possible that the antibody cross-reacted with similar proteins, hence binding different epitopes than the intended GFAP ones [24]. Interestingly, in this given study, TBI cases were more likely to contain GFAP-positive neuronal deposits, similar to the increased findings in AD brains [28] and, therefore, could be a hint for the trauma-associated neuronal damage. Alternatively, it could be suspected that the observed neuronal staining reflects lipofuscin, as mentioned previously; in this study, no lipofuscin quenching was performed due to the young age of the tested individuals. A proteomics study was able to detect GFAP as a major lipofuscin protein in humans, but not in rats [29]. In addition, lipofuscin was described to have a yellow–brown or translucent appearance under visible light [30]. However, an age-related increase would be expected for lipofuscin [31], which was not observed for the neuronal GFAP staining in this study. Furthermore, the increase of GFAP-positive staining in cases with traumatic brain injury could reflect an increase in lipofuscin and, therefore, an accelerated neurodegeneration following a traumatic brain injury. The former correlation was already described in several studies before [32].

A third possible explanation is the probability of metabolic alterations, such as acidosis [33] of brain tissue after severe traumatic events. The authors hypothesize that this might alter epitopes of intraneuronal proteins, thereby increasing the likelihood of a non-specific antibody binding. However, the altered neuronal homeostasis and its respective impact on neuronal proteins might just lead to a non-specific binding of secondary antibodies, if the vital neuron was exposed to the metabolic change for at least 2 h.

### Indicators and potential explanations for a neuronal accumulation of GFAP

GFAP is known to be a glia-specific biomarker [17, 34]. Being an intermediate filament, it is thought to contribute to cell stability [24]. Furthermore, recent studies determined GFAP plays an important role in myelination, synaptic plasticity, scar formation or the integrity of the blood–brain barrier [35–38]. Studies using a targeted deletion of the GFAP gene in embryonic stem cells creating GFAP knockout mice failed to distinguish a specific phenotype [38–40]. However, immunopositivity for GFAP in neurons was observed in pathologically altered human brain tissue, which was observed at the protein level [22, 28, 41], as well as at the RNA level [23]. Furthermore, patients suffering from AD presented out-of-frame splice forms of GFAP mRNA [28], which might result in impaired protein interaction [24]. A potential cell damage or stress might be visualized by the here observed satellitosis, which surrounded GFAP-positive labelled neurons. The neuronal origin of these immunopositive cells was proven by the double immunolabelling with the neuronal marker NeuN. The current study observed the potential immunopositivity for GFAP in few scattered neurons, rather than in all neurons of the investigated area. However, this may be due to GFAP being present in most but not all neuronal cell types, as shown with NeuN [42]. The authors hypothesize that the former might reflect an adaptation to a decreased energy supply following the traumatic event and provide an advantage to survive an acute stressful situation. The adaptation mechanism might be similar to the proposed temporary retro-differentiation of the neurons of AD patients to a GFAP-positive precursor, which differentiate into neurons after the stress survival [28].

GFAP is the major intermediate filament protein in the adult brain and characteristic for mature astrocytes. During development, GFAP is expressed by radial glia. These bipolar cells are located in the ventricular zone and have the ability to act as neural stem cells [43–45]. However, this expression only lasts during gestation. In contrast, neural precursor cells of the subventricular zone display an expression of GFAP [46, 47], which persists into adulthood [48]. With regard to TBI, immunopositivity for GFAP in neurons were detected in the CA2, CA3 and CA4 regions of the HC previously and were thought to be the result of brain swelling and brain stem haemorrhages [22]. The former study also used immunofluorescence to highlight GFAP-positive staining in neurons [22]. The results confirm the previous observation of hippocampal immunopositivity for GFAP in neurons following traumatic brain injuries [22]. Furthermore, immunopositivity for GFAP in neurons was also observed in the PCZ and the CB ipsilateral to the traumatic brain injury. Remarkably, immunopositivity for GFAP in neurons was absent in all the investigated CLCs, but was observed in the CB only in prefrontal contusions. Therefore, if the given study truly depicts intraneuronal GFAP, the data presented in this study supports that immunopositivity for GFAP in neurons occur in an area adjacent and ipsilateral to the contusion, except for some Purkinje neurons. This implies that Purkinje neurons might be more susceptible to show GFAP-positive staining in contrecoup injuries compared to cerebral neurons. However, this observation should be confirmed in a future study with a larger sample size, in order to determine its statistical significance. Contrary to the neuronal observations, the number of GFAP-positive astrocytes was shown to increase in the cerebral cortex contralateral to the contusion [5]. Only one of the 16 controls presented immunopositivity for GFAP in neurons, and this was located in the CB. Therefore, it can be hypothesized that either a small number of immunopositivity for GFAP in neurons may be expected in the CB in an atraumatic patient cohort or the person had an unknown history of a traumatic brain injury during their lifetime, or undiagnosed brain injury at death. Also, as all control cases died from sudden cardiovascular diseases, the occurrence of GFAP in neurons might be related to this disorder.

The results of this study revealed that neuronal immunopositivity for GFAP is influenced by the trauma survival time. Immunopositivity for GFAP in neurons was only observed in subacute and delayed TBIs, corroborating previous observations on immunopositivity for GFAP in hippocampal neurons [22]. This was explained by an induced expression of GFAP or a structurally related protein that is stained by the anti-GFAP immunolabelling, as well as a consequence of post-traumatic brain swelling [22]. Similarly, S100 + in neurons was mainly detected in subacute and delayed TBIs previously, with only few positive cases in acute deaths [12, 13]. Several studies and the GFAP expression pattern in certain neuronal stem cells provide evidence of the neuronal ability to express mRNA for GFAP [28, 46]. Recently, a study researching the effects of traumatic brain injury using scRNA sequencing showed an increase in neuronal GFAP expression following a traumatic event in rodents ([23] Supplemental Data). This given study reveals that supratentorial neuronal GFAP can be expected 2 h after the traumatic event, at the earliest. It has to be noted that the post-injury behaviour of the neuronal plasma membrane is not fully understood to date. It is also unclear whether GFAP with a molecular size of 50 kDa [49] can pass the damaged neuronal plasma membrane, as it is 40 kDa larger in size of the molecule that was shown to pass the disrupted membrane, previously [50]. GFAP concentrations in the cerebrospinal fluid and serum were shown to significantly increase immediately following the traumatic event [10]. However, in this study, no statistically significant changes with increasing survival times were observed [10].

### Implications of stated results for forensic questions

With regard to forensic questions such as cause of death determination, time since death estimation or survival time estimation, the usability of the findings presented in this study is limited. The GFAP immunolabelling of neurons in patients suffering from AD [28] or tuberous sclerosis [41] highlights that immunopositivity for GFAP in neurons is not TBI-specific. At most, immunopositivity for GFAP in neurons indicates a pathological state of the brain, which does not allow for a distinct cause of death determination. The immunopositivity for GFAP in neurons lacks useful information for forensic time since death estimations as the PMI did not significantly correlate with the presence of immunopositivity for GFAP in neurons in this study. However, the presented findings may be useful for TBI survival time estimations in certain cases. If a GFAP immunostained neuron is present in the PCZ or the HC, the survival time after the traumatic brain injury was likely exceeding 2 h. However, it also needs to be mentioned that the “minimum survival time” determination based on supratentorial immunopositivity for GFAP in neurons can be biased, if the deceased suffered from neurodegenerative diseases. The absence of immunopositivity for GFAP in neurons lacks useful information on the survival time as an absence of neuronal immunopositivity for GFAP is the “expected” staining result and was found regardless of the survival time or the mechanism of death.

## Limitations

Firstly, this study was limited in sample size, which affected the stated results. An increase of the case numbers could either lead to an increase of the correlation coefficient, which would strengthen the correlation or further decrease the *P*-value, which would just reassure us of the weak correlation [51, 52]. Hence, distinguishing between the mere statistical relation and the actual clinical value is necessary. Secondly, a potential degradation of GFAP has previously been reported when samples were stored for long periods [5]. However, in this study, the positive immunoreaction for GFAP in neurons did not correlate with the PMI on a statistically significant level, with the given sample size. Thirdly, single-cell immunopositivity for GFAP in neurons might have been missed during the here performed counting, which might have led to an underestimation of the stated immunopositivity for GFAP in neurons. Fourthly, immunopositivity for GFAP in neurons might not have been stained with the here applied methodology for an unknown reason. Thus, it is important to state that the absence of immunopositivity for GFAP in neurons in the here stated GFAP-negative brain samples does not imply to be the proof of any neuronal GFAP absence. Fifthly, samples were paraffin-embedded within 1–3 weeks after being formalin-fixed. This difference in formalin storage time might have impacted the semiquantitative evaluation of the given study. Future studies should use cryosectioning to evaluate whether the here observed immunopositivity for GFAP was related to the formalin storage. Furthermore, this study lacks a distinction of the particular GFAP isoforms [53–55]. A more detailed investigation would be needed to thoroughly discuss the appearance and meaning of possible GFAP-positive staining in neurons.

## Conclusions

This study provides evidence that a TBI-associated neuronal positivity for GFAP is indeed a staining artefact; however, an absence of post-traumatic neuronal GFAP cannot be confirmed. Regardless of the particular mechanism, this study revealed that the artefact/potential neuronal immunopositivity for GFAP is a global, rather than a regional brain phenomenon and might be useful for determining a minimum TBI survival time, if certain exclusion criteria are strictly respected.
